# An Exclusion Zone for Ca^2+^ Channels around Docked Vesicles Explains Release Control by Multiple Channels at a CNS Synapse

**DOI:** 10.1371/journal.pcbi.1004253

**Published:** 2015-05-07

**Authors:** Daniel Keller, Norbert Babai, Olexiy Kochubey, Yunyun Han, Henry Markram, Felix Schürmann, Ralf Schneggenburger

**Affiliations:** 1 Blue Brain Project, École Polytechnique Fédérale de Lausanne (EPFL), Lausanne, Switzerland; 2 Laboratory of Synaptic Mechanisms, Brain Mind Institute, École Polytechnique Fédérale de Lausanne (EPFL), Lausanne, Switzerland; Imperial College London, UNITED KINGDOM

## Abstract

The spatial arrangement of Ca^2+^ channels and vesicles remains unknown for most CNS synapses, despite of the crucial importance of this geometrical parameter for the Ca^2+^ control of transmitter release. At a large model synapse, the calyx of Held, transmitter release is controlled by several Ca^2+^ channels in a "domain overlap" mode, at least in young animals. To study the geometrical constraints of Ca^2+^ channel placement in domain overlap control of release, we used stochastic MCell modelling, at active zones for which the position of docked vesicles was derived from electron microscopy (EM). We found that random placement of Ca^2+^ channels was unable to produce high slope values between release and presynaptic Ca^2+^ entry, a hallmark of domain overlap, and yielded excessively large release probabilities. The simple assumption that Ca^2+^ channels can be located anywhere at active zones, except below a critical distance of ~ 30 nm away from docked vesicles ("exclusion zone"), rescued high slope values and low release probabilities. Alternatively, high slope values can also be obtained by placing all Ca^2+^ channels into a single supercluster, which however results in significantly higher heterogeneity of release probabilities. We also show experimentally that high slope values, and the sensitivity to the slow Ca^2+^ chelator EGTA-AM, are maintained with developmental maturation of the calyx synapse. Taken together, domain overlap control of release represents a highly organized active zone architecture in which Ca^2+^ channels must obey a certain distance to docked vesicles. Furthermore, domain overlap can be employed by near-mature, fast-releasing synapses.

## Author Summary

Ca^2+^ channels provide the rise in intracellular Ca^2+^ concentration necessary to initiate the membrane fusion of transmitter—filled vesicles at synapses. Because Ca^2+^ diffuses away from Ca^2+^ channels, the distance between Ca^2+^ channels and vesicles on the range of tens of nanometers is a crucial determinant of the vesicle fusion probability. However, there is still little experimental evidence on how Ca^2+^ channels and vesicles co-localize in the nanospace of a single synapse. We show by computational modelling that the channels should be located at some distance to vesicles (~ 30 nm), to allow for release control by several channels, a release mechanism found at many synapses. In realistic synapses with a high density of docked vesicles, this translates into a likely localization of Ca^2+^ channels at membrane sites not occupied by docked vesicles. Thus, we present a computational model of how Ca^2+^ channels can be localized in an active zone with several docked vesicles, to enable control of release by several Ca^2+^ channels.

## Introduction

Transmitter release at CNS synapses happens at active zones of sub-micrometer dimensions, which harbor docked vesicles and vesicle fusion proteins, as well as presynaptic scaffold proteins and voltage-gated Ca^2+^ channels [1]. The number, and distance of Ca^2+^ channels to readily-releasable vesicles are crucial for determining release probability, because the Ca^2+^ signal generated by a single open Ca^2+^ channel drops off steeply with distance [2–5]. There is, however, only sparse morphological information on the co-localization of individual vesicles and Ca^2+^ channels at synapses. For this reason, Ca^2+^ channel—vesicle coupling distances have often been inferred from functional measurements.

One approach to functionally assess the number of Ca^2+^ channels controlling release utilizes the high intrinsic Ca^2+^ cooperativity of release [6–8]. In experiments in which the number of open Ca^2+^ channels is varied in presynaptic voltage-clamp experiments, the slope value in plots of transmitter release vs integral Ca^2+^ influx in double-logarithmic coordinates (which we will call the "Ca^2+^ current release cooperativity" or simply "slope value") can inform about the number of Ca^2+^ channels involved in release control [9–13]. A large Ca^2+^ current release cooperativity close to the intrinsic Ca^2+^ cooperativity indicates that Ca^2+^ signals from individual channels mix to produce a graded Ca^2+^ signal at each docked vesicle. Conversely, a slope value close to one indicates that a few, or a single Ca^2+^ channel control the release of a given docked vesicle [9–13]. The diffusional distance between Ca^2+^ channels and vesicles can also be probed by the slowly binding Ca^2+^ chelator EGTA, which affects Ca^2+^ signals only at some distance from the Ca^2+^ source [14–17]. Experiments with these functional approaches have shown different coupling regimes at different CNS synapses. At the calyx of Held synapse which is amenable to direct presynaptic patch-clamp experiments, release is sensitive to EGTA at low millimolar concentrations, and high slope values are observed, indicating domain overlap control of release [18,19]. However, Ca^2+^ channel—release coupling was later shown to be developmentally regulated, such that brief presynaptic Ca^2+^ currents become more effective in causing transmitter release [12,20–22].

Electron microscopy (EM) has shown that active zones of various types of CNS synapses are small (~ 0.1 μm^2^), and contain a high density of docked vesicles [23–26]. The development of EM freeze fracture replica labeling techniques has enabled the visualization of ion channels and receptors in *en-face* views at nanometer resolution [27]. Using this method, multiple stripe—like clusters of P/Q—type Ca^2+^ channels (Ca_V_2.1 subunits) have been observed at hippocampal synapses [28]. However, with freeze fracture replica labeling, the position of docked vesicles cannot be visualized, and antibody-labelled particles do not necessarily overlap with the entire population of functionally active Ca^2+^ channels. Therefore, despite important advances in ultrastructural methods, the functionally relevant positions of Ca^2+^ channels and vesicles on the nanometer scale are still uncertain.

Here, we use stochastic MCell modelling of Ca^2+^ influx through individual Ca^2+^ channels at EM—reconstructed active zones, to explore how Ca^2+^ channels might be positioned to produce domain overlap control of release. The position of docked vesicles was fixed by previous EM reconstructions of single active zones [29]. Other model parameters, like presynaptic Ca^2+^ channel gating and—conductance [18,19,30–32], Ca^2+^ buffering [33–35], and the intracellular Ca^2+^—sensitivity of vesicle fusion [7,8,22,36–39] were constrained by previous measurements at the calyx of Held and at other large model synapses. We find that random placement of Ca^2+^ channels leads to an excessively high release probability and low slope values. In order to enable domain overlap control of release, we rather need to assume that Ca^2+^ channels are kept at some distance from vesicles. We also show experimentally that the Ca^2+^ current—release cooperativity stays high after the onset of hearing in mice, suggesting that despite a characteristic developmental tightening in the Ca^2+^ channel vesicle co-localization [12,20–22], fast release in more mature calyx synapses continues to be controlled by several Ca^2+^ channels.

## Results

### Geometric distribution of docked vesicles at the active zone of calyx synapses

**Fig 1:**
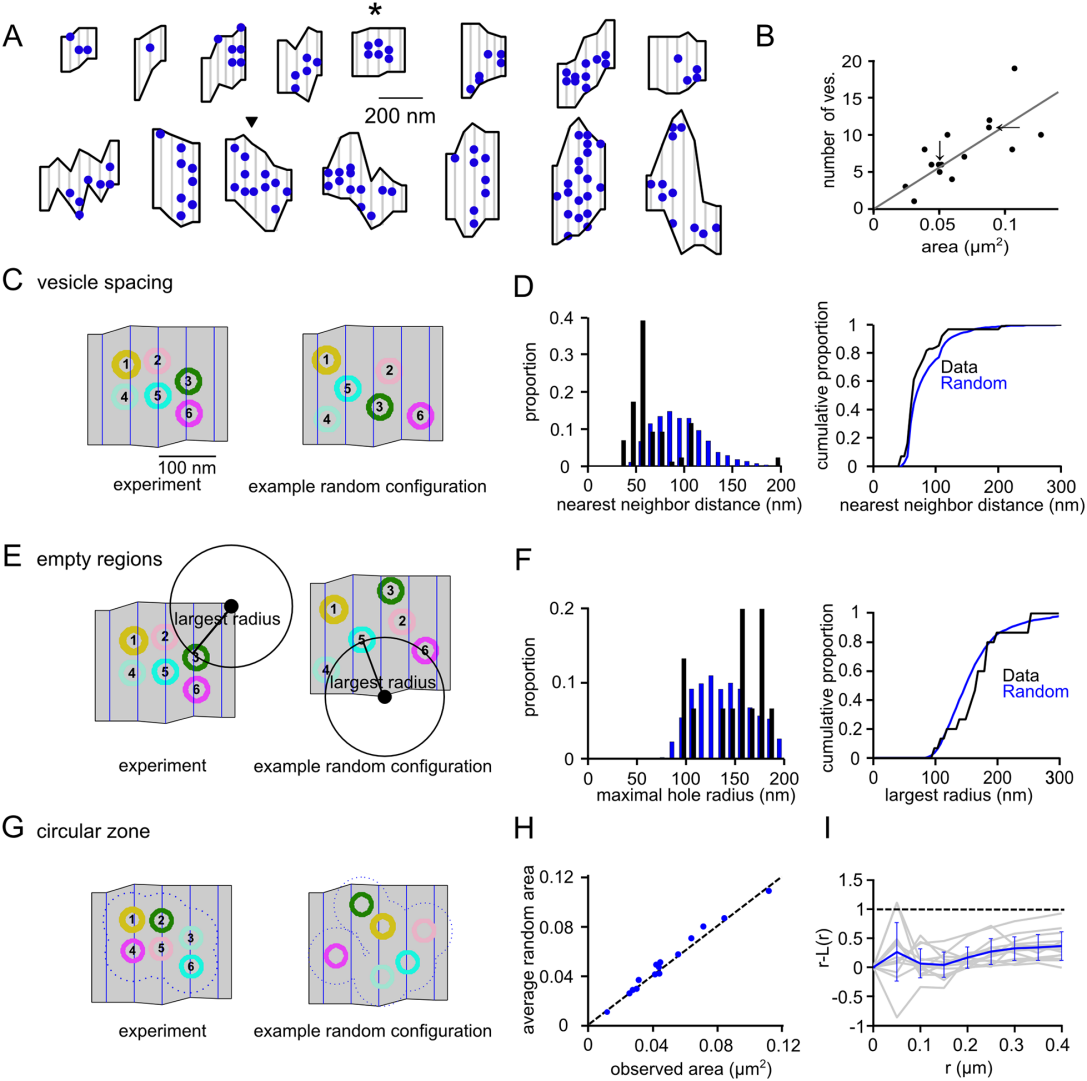
Analysis of the spatial distributions of docked vesicles at the mouse calyx of Held synapse. (**A**) Top-down views of n = 15 completely reconstructed active zones from a P11 mouse [29], with indicated docked vesicle positions (blue). Star- and triangle symbols indicate active zones #5 and # 11 used for simulations here (see below, Figs 2–5). (**B**) Plot of the number of docked vesicles versus active zone area. Note the reasonable correlation (r = 0.74). The values for active zones # 5 and # 11, used for subsequent simulations, are highlighted by the vertical and horizontal arrow, respectively. (**C**) Analysis of the clustering of docked vesicles based on the nearest neighbor distance between vesicles. *Left image* shows the experimentally determined docked vesicle localization at active zone # 5 as an example; *right image* shows a single spatial sample of randomly localized docked vesicles. (**D**) Histograms of the distribution of distances for the random case (blue bars; n = 4800 repetitions for each active zone) and for the experimentally observed case (black; average ± S.D. 67.6 ± 19.0 nm; n = 88 docked vesicles from all N = 15 active zones). The experimental value was slightly smaller than the simulated random case (blue bars; 79.8 ± 20.2 nm; average ± S.D.; p = 8.5 10^–4^; paired t-test), which indicates some clustering of docked vesicles. The right panel shows cumulative histograms. (**E**) Analysis of the clustering of docked vesicles based on a largest radius method. For each active zone, we computed the maximal radius of a circle whose center was in the active zone and did not overlap any vesicles (*left image*, for the example active zone). This was compared to distributions of circle radii for the same active zone, but with randomly placed vesicles (*right image*, example of a random seed of vesicle localization). (**F**) The distribution of circle radii when the vesicles are randomly distributed within all active zones (blue; n = 150 independent seeds for each active zone; 151.8 ± 39.2 nm), compared to the data distribution (black; 160 ± 44.7 nm). The cumulative distribution for the data (black) and random vesicle arrangements (blue; all 15 active zones) are similar. The mean of all 15 active zones for which the random configurations were tested, was statistically indistinguishable from the data mean (paired t-test; p = 0.25). (**G, H**) Analysis of the clustering of docked vesicles based on comparing areas included within a 30 nm exclusion zone. For each active zone, docked vesicles were placed in random positions, and the area within a 30 nm exclusion zone was calculated. The mean of the areas calculated from random placements (n = 3000 for each active zone) was plotted against the measured exclusion zone area for all N = 15 active zones (**I**). This follows a line with slope of 1, indicating little clustering at this exclusion zone size. (**I**) We used Ripley’s K function as a metric for detecting deviations from spatial homogeneity [40]. The quantity r-L (r) was computed (see S1 Text), and then plotted versus radius and normalized to the 99% confidence interval (dashed horizontal line). The mean for all active zones is shown in blue, while individual active zones are shown in gray. The plotted measure is below the 99% confidence interval for all radii tested, indicating no significant vesicle clustering.

We wished to explore the possible placement of Ca^2+^ channels compatible with domain overlap control of release. For this, it is important to know the distribution, and density of docked vesicles typically observed at active zones. To determine these parameters, we analyzed a sample of n = 15 reconstructed active zones of calyx synapses of a P11 wild-type mouse [29] (Fig 1A). In this sample, the average surface area of active zones was 0.07 ± 0.03 μm^2^, with an average density of docked vesicles of 110 ± 40 ves / μm^2^ (n = 15). Across individual active zones, the number of docked vesicles and active zone surface were correlated (r = 0.74; Fig 1B), indicating that large active zones tend to harbor more docked vesicles [20,23].

Visual inspection of the active zone maps with their docked vesicles (Fig 1A) suggests that some active zones show sub-areas where docked vesicles are sparse. We will show below that in domain overlap control scenarios, such areas might be preferentially occupied by Ca^2+^ channels. To investigate whether such void spaces could arise randomly, we compared experimentally observed vesicle distributions with the same parameters derived from random x-y placements of vesicles at a given active zone (Fig 1C, 1D, 1E, 1F, 1G and 1H). Analyzing nearest neighbor distances, and the largest hole radii capable to fill empty spaces within active zones suggested that the observed vesicle positions were close to the random case (Fig 1C and 1D, Fig 1E and 1F, respectively). Similarly, calculating the summed area extending 30 nm away from the edge of docked vesicles resulted in similar areas for randomly distributed vesicles, and for the docked vesicles found in the data set (Fig 1G and 1H). Finally, we used Ripley’s K function test, a metric for detecting deviations from spatial homogeneity [40] (see S1 Text). It gave values within the 99% confidence interval for all distances tested (Fig 1I), indicating no significant vesicle clustering in the reconstructed active zones. Thus, there was overall no strong vesicle clustering in the reconstructed active zones.

### Random placement of Ca^2+^ channels does not predict key aspects of domain overlap release control

**Fig 2:**
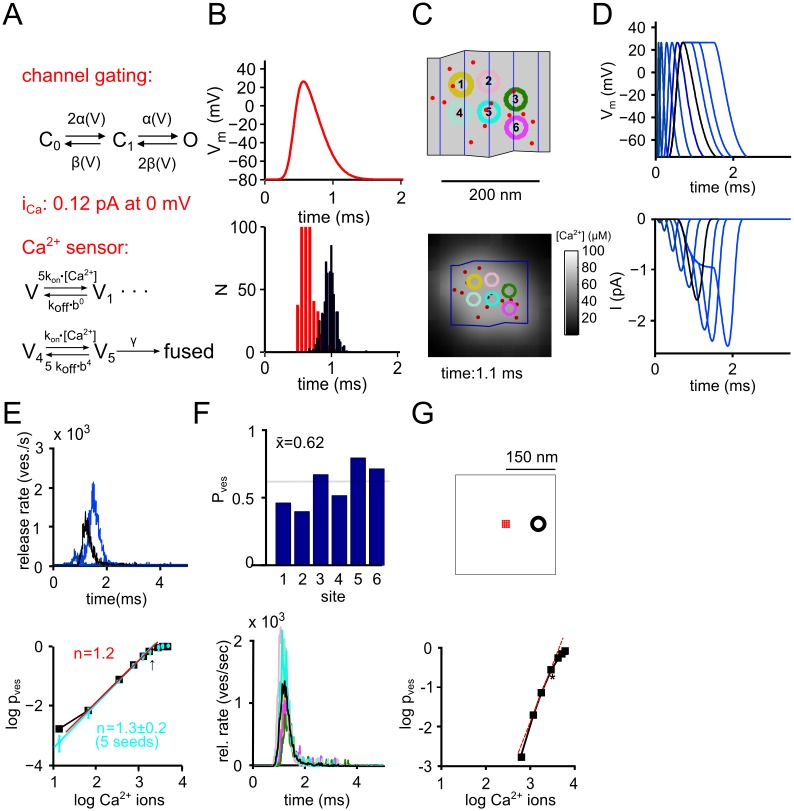
Randomly distributed Ca^2+^ channels in a realistic active zone cannot explain domain overlap control of release. (**A**) Illustration of the elements of the computational model (see main text, and Material and Methods for details). (**B**) The standard AP used for the simulation (upper panel), and the distribution of Ca^2+^ channel opening and closing times (*red* and *black* histogram bars, respectively). (**C**) Illustration of one spatial sample in which n = 14 Ca^2+^ channels (red dots) were placed randomly in active zone #5 (see Fig 1A; star symbol). The bottom panel shows a map of the near-membrane [Ca^2+^]_i_ at 1.1 ms, close to the peak of the Ca^2+^ current. (**D**) APs used for simulating the "Ca^2+^ current—release cooperativity" (*top*); and the resulting Ca^2+^ currents (*bottom*). The standard AP, and the resulting active zone Ca^2+^ current are shown by black traces. (**E**) Simulated transmitter release rates (*top*) for three selected AP widths (black trace corresponds to AP with standard width), and double-logarithmic plot of average vesicular release probability (p_ves_) versus Ca^2+^ influx for the different AP widths (*bottom*). Note the low slope value of n = 1.3 ± 0.2 (blue symbols are average data points of n = 5 independent spatial seeds), indicating that a random localization of Ca^2+^ channel is unlikely to explain domain overlap control. (**F**) Plot of vesicular release probability (p_ves_) at each release site (*top*), and simulated release rates for the n = 6 vesicle docking sites (*bottom*; same color codes as in C). Note the unrealistically high values of p_ves_. (**G**) Simulations with a single supercluster of n = 14 channels (red dots, *top*) and a single vesicle placed at 100 nm distance (black circle, *top*). Note the high slope which can be reached in this arrangement (*bottom*; slope value = 2.8).

We next investigated whether random placement of Ca^2+^ channels in a realistic active zone with several docked vesicles can predict release control by several channels. We developed an MCell based model which incorporated individual Ca^2+^ channels with realistic gating and permeation properties [19,31,41] (Fig 2A). The model contained several intracellular Ca^2+^ buffers, and each vesicle had a Ca^2+^ sensor for vesicle fusion according to a highly non-linear five-site ion binding mechanism [7,8] (Fig 2A). The parameters of the Ca^2+^ sensor model were determined in several independent Ca^2+^ uncaging studies [7,8,22,36,37], and were fixed to the values reported in one previous study [37]. Several other parameters, including the width of the presynaptic AP (Fig 2B) were also tightly constrained by previous biophysical experiments at the calyx of Held synapse (see Materials and Methods, and S2 Text). The Ca^2+^ channel density was set at a value of 280 /μm^2^ (ref. [28,41]).

For most simulations, we used single representative active zones drawn from the sample of reconstructed active zones. We started with active zone #5 (see Fig 1A, star symbol) which has an area of 0.05 μm^2^ (slightly smaller than the mean), and a vesicle docking density of 120 ves / μm^2^, representative of the sample (Fig 1B, vertical arrow). We placed n = 14 Ca^2+^ channels randomly in this active zone (Fig 2C), corresponding to a Ca^2+^ channel density of 280 /μm^2^. We simulated a "Ca^2+^ current—release cooperativity" experiment by driving release with APs of different widths (Fig 2D), and plotted the resulting average vesicle release probability, p_ves_, as a function of Ca^2+^ entry in double-logarithmic coordinates (Fig 2E). This yielded a slope value of only 1.3 ± 0.2 (n = 5 independent random spatial samples), far smaller than a value of ~ 3–3.5 expected for domain overlap control of release (see below). In addition, the average p_ves_ in response to the standard AP was 0.66 ± 0.18 (Fig 2F), much higher than experimental estimates (~ 0.1; ref. [8]). As a control, we placed n = 16 Ca^2+^ channels in a tight cluster at 100 nm from a single vesicle. This resulted in a slope value of 2.8 (Fig 2G), close to the maximal value of 3.5 which can be achieved for kinetic models with 5 Ca^2+^ binding sites [11,42]. Thus, these simulations suggest that a random placement of Ca^2+^ channels is unable to produce high Ca^2+^ current—release cooperativities close to the experimentally observed values.

### Assuming an exclusion zone around docked vesicles explains release control by several Ca^2+^ channels

**Fig 3:**
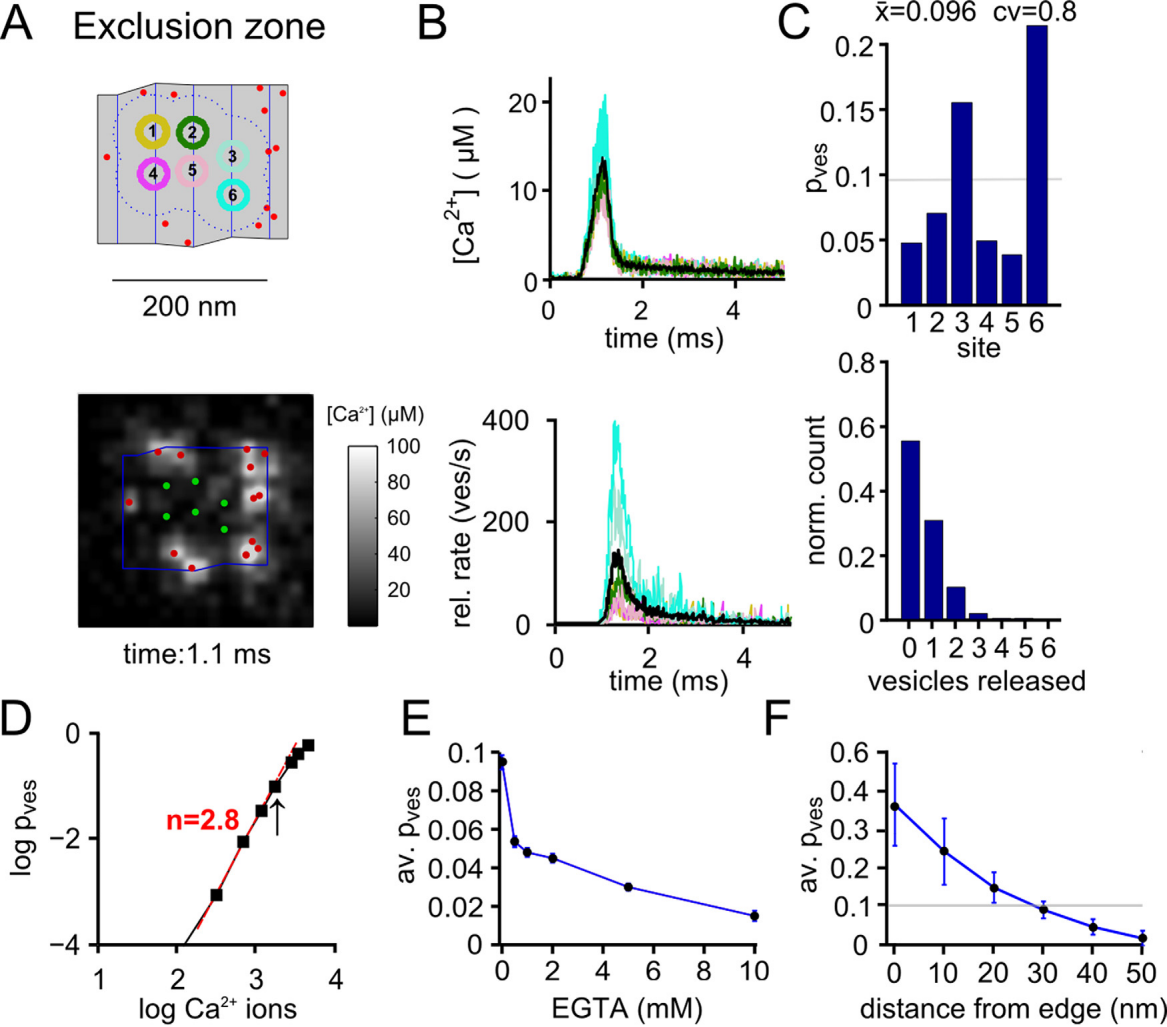
Keeping Ca^2+^ channels at some distance from docked vesicles enables domain overlap control of release. (**A**) Illustration of one spatial sample of n = 14 Ca^2+^ channels located in active zone # 5 in the "exclusion zone" model (minimal distance from docked vesicle edge; 30 nm). *Bottom* shows the near-membrane [Ca^2+^]_i_ at 1.1 ms. (**B**) [Ca^2+^]_i_ reached at each docked vesicle during the standard AP (*top*), and the resulting release rates (*bottom*). (**C**) Plot of p_ves_ for the individual vesicle docking sites (*top*), and distribution of the number of released vesicles over the entire active zone (bottom), both computed for n = 8000 trials with a standard AP. (**D**) Double-logarithmic plot of p_ves_ versus Ca^2+^ entry for the simulation of the Ca^2+^ current—release cooperativity experiment. Note the high slope of 2.8 predicted for this spatial sample (see A; n = 2000–4000 repetitions for each AP width). On average, a slope value of 2.8 ± 0.1 resulted (n = 4 independent spatial samples). (**E**) Simulation of the EGTA—sensitivity of release driven by the standard AP. Note the biphasic sensitivity of release to EGTA, as observed experimentally [15]. (**F**) Dependence of p_ves_ on the exclusion zone size, measured as the minimal distance from vesicle edge. Note that an exclusion zone size of 30 nm produces a physiologically plausible p_ves_ of 0.1 (grey line).

It has been shown that a large number of Ca^2+^ channels (> 10–20) is needed to produce high Ca^2+^ current—release cooperativity [11]. If such a large number of channels controls release of a given vesicle with roughly equal strength, then it seems plausible to assume that the channels must be located at some distance to vesicles. A simple implementation of such a rule in a field of randomly placed docked vesicles at relatively high density (Fig 1) is to assume an exclusion zone around each docked vesicle; a zone into which Ca^2+^ channels cannot enter. We implemented this exclusion zone rule by assuming that Ca^2+^ channels can be located anywhere at the active zone, but not below a distance of 30 nm (Fig 3A). When the exclusion zone model was driven with a standard AP, brief local [Ca^2+^]_i_ transients with amplitudes of 10–20 μM resulted at individual docking sites (Fig 3B), similar to results based on back-calculation from Ca^2+^ uncaging data [7,8]. The vesicular release probabilities p_ves_ were variable across individual docked vesicles, with a realistic average value of ~ 0.1 (Fig 3C, upper panel). The distribution of the number of released vesicles showed that most trials (~ 60%) led to release failures at an active zone (Fig 3C), again in agreement with previous estimates [43].

We then simulated the Ca^2+^ current—release cooperativity experiment using different AP widths, and found that the exclusion zone model with a distance of 30 nm produced high values of Ca^2+^ current—release cooperativity (n = 2.8 ± 0.2; n = 4 independent spatial samples; Fig 3D). We attribute this to the fact that multiple Ca^2+^ channels (n = 6–14) should influence the release of a given vesicle. Thus, assuming that Ca^2+^ channels can be placed anywhere except at very close distances to docked vesicles (~ 30 nm) predicts a high Ca^2+^ current—release cooperativity, a hallmark of release control by several Ca^2+^ channels [9–13,22].

We also simulated the sensitivity of release to the slow Ca^2+^ buffer EGTA in the exclusion zone model (Fig 3E). For this, release in response to single standard APs was modelled in the presence of EGTA, added to the Ca^2+^ buffers present in the standard model (see Materials and Methods). These simulations showed that 0.5 mM EGTA suppressed release by ~ half, with an overall biphasic concentration—dependence of EGTA (Fig 3E), similar to what was found at the calyx of Held synapse and at cortical synapses of young rats [15,18]. Finally, we tested the influence of the exclusion zone width on release probability p_ves_, and found an inverse relation of p_ves_ with exclusion zone distance, with a value for p_ves_ of 0.096 reached at 30 nm (Fig 3F, horizontal line). We conclude that the exclusion zone model can reproduce many features of domain overlap control of release reported in previous functional studies.

### A single supercluster of Ca^2+^ channels produces large p_ves_ heterogeneity

**Fig 4:**
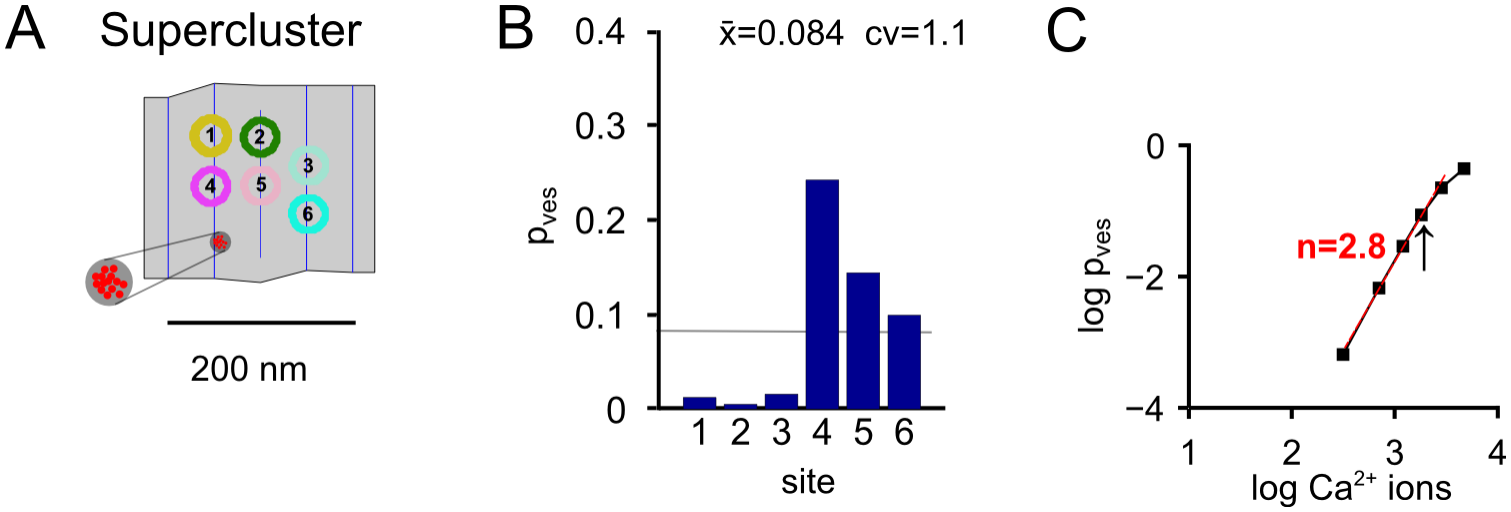
Placing all Ca^2+^ channels in a single supercluster produces high heterogeneity of p_ves_. In these simulations, we assumed that all Ca^2+^ channels (n = 14 for this active zones) were localized in a single tightly spaced cluster [11], using again the example active zone # 5. (**A**) x-y arrangement of vesicles and Ca^2+^ channels. (**B**) Values of p_ves_ for individual vesicle docking sites in response to stimulation with a standard AP. The average value and c.v. are indicated. (**C**) Double-logarithmic plot of average p_ves_ versus Ca^2+^ influx, indicating that as expected, the supercluster model is able to produce a high Ca^2+^ current—release cooperativity [11].

In a previous model, release control by several Ca^2+^ channels was achieved by placing all channels in a single cluster [11], an arrangement which we will call "supercluster". We next tested this arrangement, by first using the docked vesicle distribution of active zone #5. The distance of the supercluster to the nearest vesicle was 30 nm (Fig 4A), which yielded an acceptable *average* p_ves_ of 0.08 (Fig 4B) and a high Ca^2+^ current—release cooperativity (2.8 ± 0.1; n = 4 independent spatial samples; Fig 4C). A characteristic property of the supercluster arrangement is, however, a very large heterogeneity between p_ves_ values for individual vesicles (range, 0.005 to 0.24; c.v. = 1.1; Fig 4B), because far—away vesicles experienced a much lower Ca^2+^ signal.

**Fig 5:**
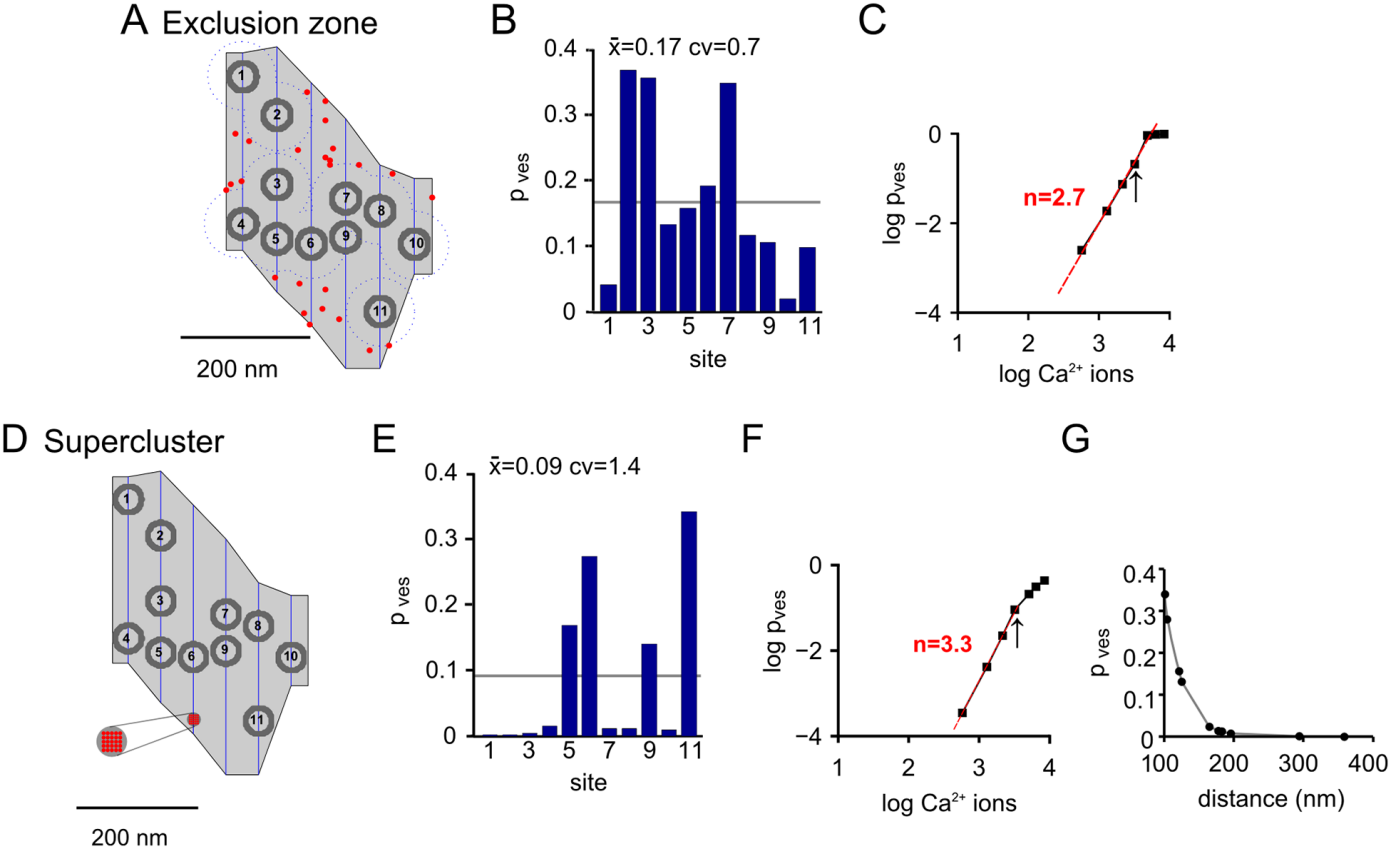
Simulations at a larger example active zone show more drastically that placement of all Ca^2+^ channels in a single supercluster produces excessive heterogeneity of p_ves_. (**A**) Active zone # 11 (see Fig 1A, triangle) was used for the simulations. We assumed the presence of n = 25 Ca^2+^ channels to maintain a constant Ca^2+^ channel density across active zones. One spatial seed is shown for an exclusion zone rule for Ca^2+^ channels (red dots) with minimal distance of 30 nm from the edge of each vesicle. (**B**) Resulting distribution of p_ves_ values in response to release triggered by the standard AP, for the spatial seed illustrated in (**A**). The number on the abscissa ("site") corresponds to the vesicle number in (**A**). (**C**) The plot of vesicular release probability versus Ca^2+^ influx, for the spatial seed illustrated in (**A**). Note the reasonably high slope value (2.7). (**D-F**) Model of the same active zone as in (**A-C**), but now with all Ca^2+^ channels (n = 25) placed in a single supercluster (red spot). Note the large heterogeneity in p_ves_ and the small values of p_ves_ for far-away vesicles (**E**). A high slope value of 3.3 was apparent in this simulation (**F**). (**G**) Dependence of p_ves_ on the distance of each vesicle from the Ca^2+^ channel supercluster.

We next compared the exclusion zone—and supercluster channel arrangements at another example active zone with larger size (active zone # 11; see above Fig 1A, triangle symbol). This active zone also had a representative density of docked vesicles (see Fig 1B, horizontal arrow). In this active zone, 25 Ca^2+^ channels were used to again yield a Ca^2+^ channel density of 280 /μm^2^. Assuming an exclusion zone with a distance of 30 nm (Fig 5A), we found a somewhat higher average p_ves_ (0.17; Fig 5B) as compared to the smaller active zone # 5 (see above; Fig 3; p_ves_ ~ 0.1). This finding, which is compatible with recent experimental findings [41], might suggest that at comparable Ca^2+^ channel densities, large active zones use Ca^2+^ more efficiently because of smaller diffusional loss of Ca^2+^ away from the active zone. However, further modelling studies are needed to investigate this mechanism in more detail. In the simulations of Fig 5A–5C using an exclusion zone distance of 30 nm, the individual p_ves_ values ranged from 0.02 to 0.36 with a c.v. of 0.7 (Fig 5B), and the Ca^2+^ current—release cooperativity was 2.7.

We next modelled Ca^2+^—release control at this large active zone assuming that all Ca^2+^ channels (n = 25) are located within a single supercluster. We placed the supercluster into a void space in the bottom half of the active zone, and varied its exact position until we achieved a reasonable average p_ves_ of 0.1 (Fig 5D and 5E). The supercluster model again yielded a high slope value (3.3; Fig 5F). However, the heterogeneity of p_ves_ was very large, with a c.v. value of 1.4. Indeed, visual inspection of this relatively large active zone shows many vesicles that are far away from the cluster (e.g. vesicles 1, 2, 7 and 8; Fig 5D). Correspondingly, these vesicles showed extremely low p_ves_ values (0, 0.0001, 0.0129, and 0.012, respectively). As expected, there was an inverse relation between p_ves_ and distance of the docked vesicle from the Ca^2+^ channel supercluster (Fig 5G). We note, therefore, that the supercluster model produces a high heterogeneity of p_ves_, especially in large active zones.

### Neither the supercluster nor the exclusion zone models predict slow SRP release kinetics

Since the two arrangements of Ca^2+^ channels predict distinct heterogeneities of p_ves_, experimental data on the heterogeneity of p_ves_ within individual active zones would be necessary to verify the supercluster versus the exclusion zone arrangement. Unfortunately, the distribution of p_ves_ within individual active zones following AP stimulation has, to our knowledge, not been estimated experimentally; a previous theoretical study has highlighted the role of p_ves_ heterogeneity for synaptic depression [44]. On the other hand, studies at the calyx using prolonged presynaptic depolarizations have revealed that release occurs in distinct kinetic phases, with time constants of ~ 2 ms and ~ 20 ms called fast- and slowly releasable pool (FRP and SRP, respectively) [45–47]. Some studies have interpreted the different release kinetics as a result of different distances of vesicles to Ca^2+^ channels (the "positional" model; [46,48,49]), but other studies concluded that SRP release results from intrinsically slower release [47,50] (see Discussion).

**Fig 6:**
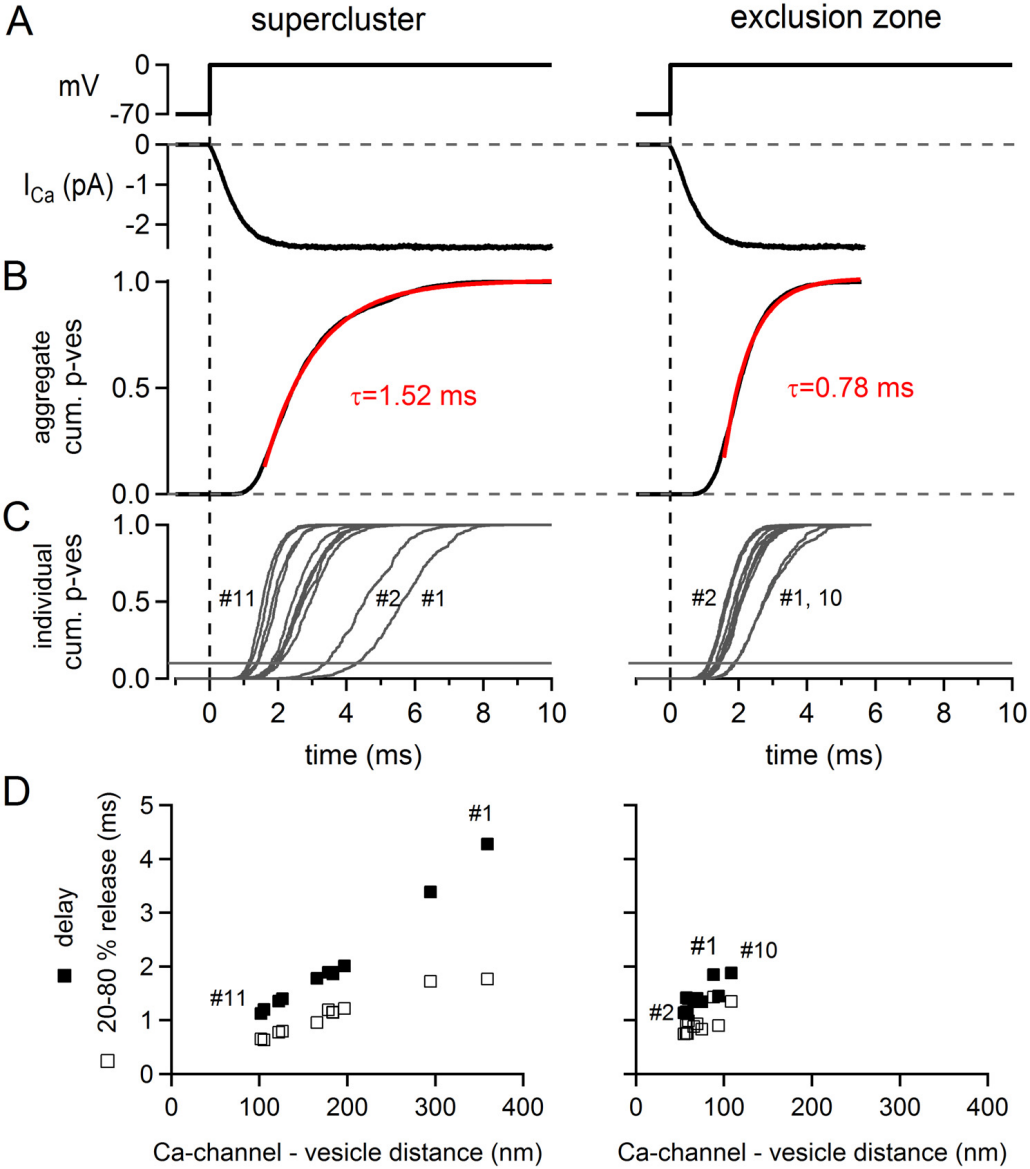
Neither the supercluster nor the exclusion zone model predict slow release in response to long depolarizations. Simulations in response to a long depolarizing step to 0 mV [45] were made both for the supercluster arrangement (*left*) and for the exclusion zone arrangement (*right*) for the large active zone (active zone # 11; see Fig 5A and 5D for the spatial arrangements). (**A**) Voltage step from -70 mV to 0 mV and resulting Ca^2+^ current for the supercluster model (left) and the exclusion zone model (*right*). (**B**) The resulting aggregate cumulative release rate, averaged over all n = 11 vesicles at this active zone. The data traces (*black*) were fitted with a single exponential with time constant of 1.52 ms (*left*, supercluster arrangement) and 0.78 ms (*right*, exclusion zone model). (**C**) The cumulative release rate traces of the individual vesicles, for the supercluster and exclusion zone model. Note the larger spread of release delays in the supercluster model (*left*) as compared to the exclusion zone model (*right*). (**D**) Analysis of the delay (time needed to reach 10% of cumulative release), and the 20–80% rise times of the cumulative release rates as a function of Ca^2+^ channel—vesicle distance. For the supercluster model (*left*), an unequivocal distance between the supercluster and each vesicle can be computed. In the case of the exclusion zone model, the distance is given as the average distance of each vesicle to the three nearest Ca^2+^ channels. In C and D, numbers indicate some relevant vesicle positions (see Fig 5A and 5D).

To further validate the exclusion zone model and the supercluster model, we modelled release in response to a prolonged presynaptic depolarization to 0 mV, using the large active zone with either the supercluster—or the exclusion zone arrangement of Ca^2+^ channels (Fig 6). To our initial surprise, both models produced fast release, without obvious SRP component (Fig 6A, 6B, and 6C). Exponential fits of the average cumulative release rates yielded time constants of 1.52 ms and of 0.78 ms for the supercluster model and the exclusion zone model, respectively (Fig 6B, red fit lines). In each case, a double-exponential function did not improve the fit (not shown). When we plotted release from the individual sites separately, it became apparent that the spread of release delays and 20–80% release times was larger for the supercluster model (Fig 6C and 6D; *left*) than for the exclusion zone model (Fig 6C and 6D; *right*). This is expected, since the heterogeneity of the Ca^2+^ channel—vesicle distances is larger in the supercluster model than in the exclusion zone arrangement (see above; Fig 5). The faster average release time constant in the exclusion zone model as compared to the supercluster model (0.8 ms versus 1.5 ms) corresponds to the lower degree of p_ves_ heterogeneity in the exclusion zone arrangement. Indeed, far-away vesicles in the supercluster arrangement have release probabilities near zero during AP stimulation (e.g. vesicles #1 and # 2; see above Fig 5D and 5G), and these vesicles are released with an additional delay of ~ 2 ms upon prolonged depolarization to 0 mV (Fig 6C and 6D).

Taken together, simulating the response to prolonged presynaptic depolarizations does not give further clues to distinguish between the two Ca^2+^ channel arrangements (exclusion zone versus supercluster), since both models were unable to predict SRP release with time constants of ~ 20 ms, as observed experimentally in immature calyx of Held synapses [45,47]. We conclude that to explain SRP release, one would either need to postulate Ca^2+^ channel—vesicle distances longer than 400 nm. Alternatively, SRP release could be caused by intrinsically slower release kinetics as suggested by previous Ca^2+^ uncaging experiments [22,47,51].

### Ca^2+^ current—Release cooperativity remains high upon initial synapse maturation

We next wanted to investigate whether transmitter release in more mature calyx of Held synapses might still be controlled in a domain overlap fashion. Previous work has shown a developmental tightening in the spatial co-localization between Ca^2+^ channels and docked vesicles, as demonstrated by an increased efficiency of small Ca^2+^ charges in inducing release [20–22], and a reduced efficiency of EGTA in suppressing release and reduced Ca^2+^ current—cooperativity values [12]. The question therefore arises whether release in more mature calyx synapses is converted into a single channel control mode or else, whether several Ca^2+^ channels still control release of a given vesicle, albeit at somewhat shorter distance.

**Fig 7:**
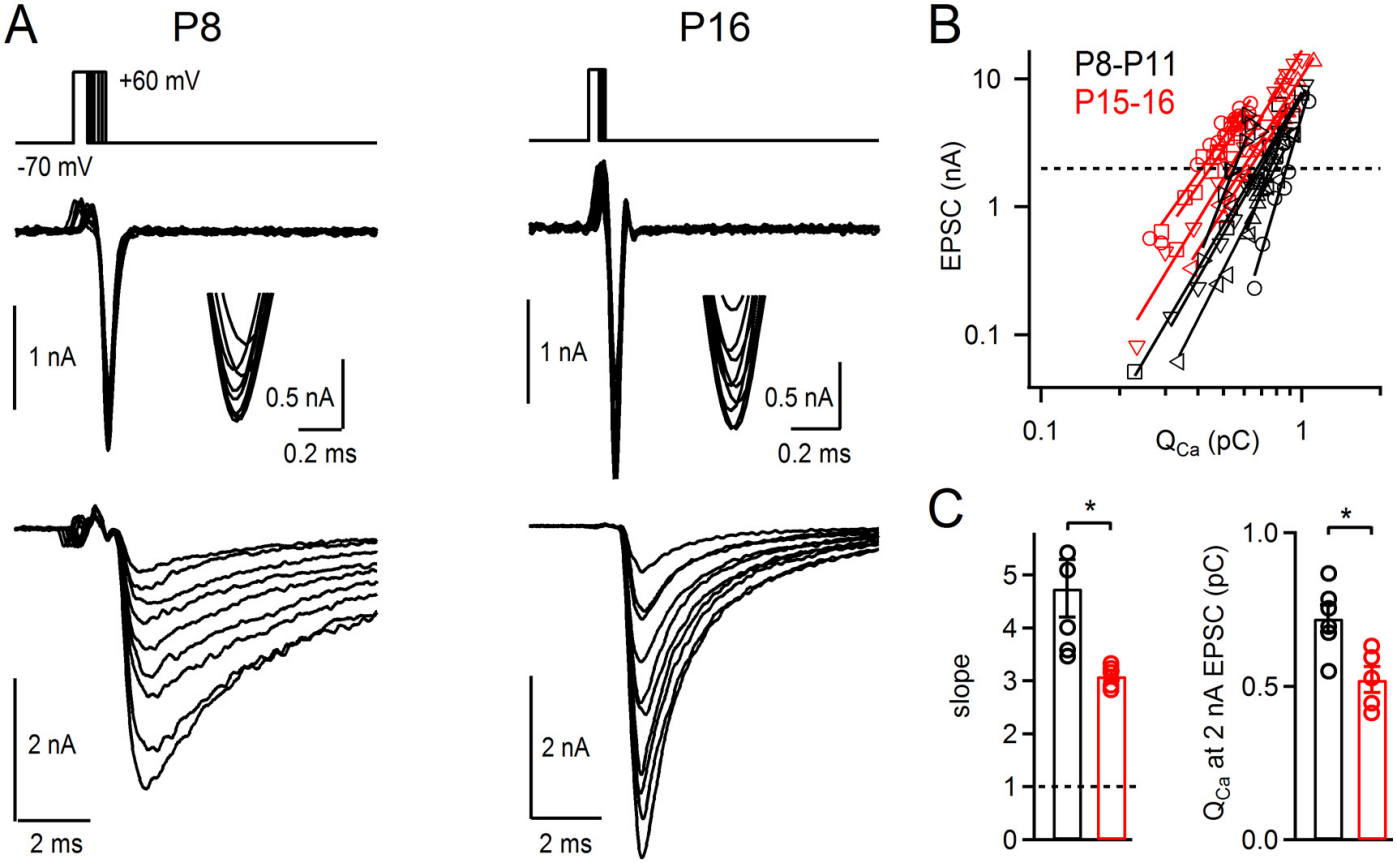
The Ca^2+^ current—Release, cooperativity stays elevated in mature calyx of Held synapses. (**A**) Representative example traces of paired pre- and postsynaptic voltage-clamp experiments at a calyx of Held synapse of a P8 mouse (*left*), and at a P16 mouse (*right*). Panels from top to bottom show the voltage-clamp protocol, the presynaptic Ca^2+^ tail current, and the resulting EPSCs. The insets show the peaks of the presynaptic Ca^2+^ tail currents at higher resolution. (**B**) Plot of EPSC amplitudes versus integrated presynaptic Ca^2+^ charge for all data (n = 6 and 5 paired recordings at P8–P11 and at P15–P16 respectively). Note the double-logarithmic coordinates, and the steep slopes but slight leftward shift in older mice (red symbols). (**C**) Individual and average values for the slopes (*left*), and for the presynaptic Ca^2+^ charge needed to evoke an EPSC of 2 nA (*right*). Although both values were significantly lower at P15–P16 (p < 0.05 for both data sets), the slope value was still high in the more mature mice, suggesting release control by multiple channels.

To investigate this issue, we made measurements of Ca^2+^ current release cooperativity at postnatal day (P) P8–P11, and in P15–P16 old mice, the oldest age group amenable to paired recordings under our conditions. We varied the number of open Ca^2+^ channels in Ca^2+^ "tail" current experiments with voltage steps of different lengths, and measured the corresponding EPSC amplitudes as a proxy of release (Fig 7A). Plotting the EPSC amplitudes versus the integral Ca^2+^ charge in double logarithmic coordinates then allowed us to measure the Ca^2+^ current—release cooperativity (slope value), and the Ca^2+^ charge (Q_Ca_) necessary to evoke an EPSC of 2 nA. The slope values were 4.75 ± 0.60 (n = 6 pair recordings) for mice at P8–P11, and 3.10 ± 0.11 (n = 5 pair recordings) for P15–P16 mice (Fig 7C). Thus, although there was a significant decrease of the slope value with development (p < 0.05), the slope of ~ 3 was still significantly higher than 1 (p < 0.001; one-sample two-tailed t-test), the value expected for the extreme case of single-channel vesicle release control [9,13]. In addition, we observed that the Ca^2+^ charge (Q_Ca_) necessary to evoke an interpolated EPSC of 2 nA was shifted to smaller values (Fig 7B and 7C; p < 0.05), similarly as reported before for the rat calyx of Held [22]. Both findings indicate a tighter coupling in the more mature mice [12,20,21]. Despite the tighter coupling, the Ca^2+^ current—release cooperativity was still high (~ 3), which indicates that several Ca^2+^ channels control the release of a given vesicle also in more mature synapses.

### Adult calyx synapses are sensitive to EGTA-AM

**Fig 8:**
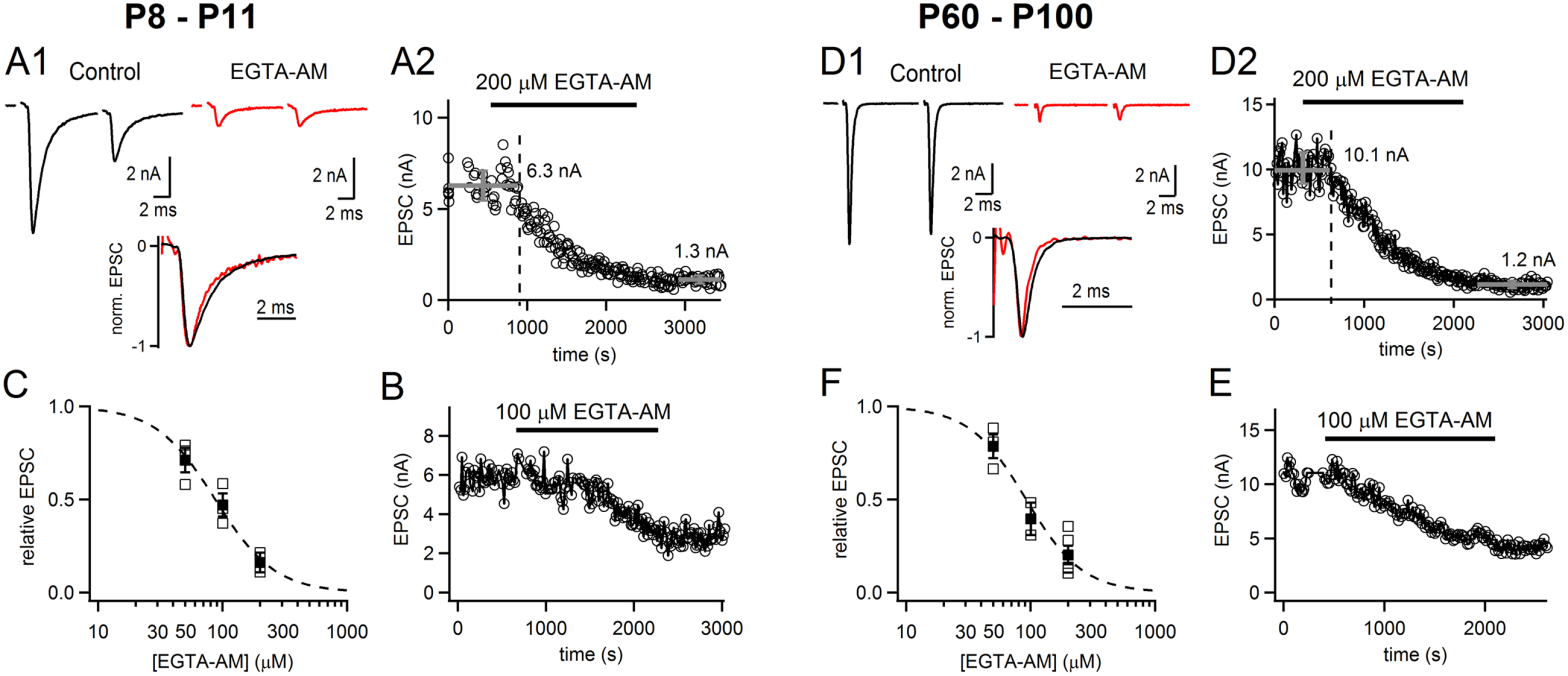
Fast transmitter release at the calyx synapse remains sensitive to the slow Ca^2+^ buffer EGTA-AM up to adulthood. (**A1**) Example EPSC traces before (*black*) and after application of EGTA-AM (*red*; 200 μM) in a P8 mouse. The inset shows the EPSC trace before and after EGTA-AM normalized to their peak amplitudes. Note the largely unchanged kinetics of the EPSCs following EGTA-AM application (*red* trace). (**A2**), Plot of EPSC amplitude versus recording time, for the same cell as illustrated in A1. (**B**) Another representative example for application of 100 μM EGTA-AM in a different recording from an immature synapse; note that 100 μM EGTA-AM suppresses EPSC amplitudes more slowly, and to a lesser final degree. (**C**), Concentration-dependence of the EPSC suppression by EGTA-AM, measured in P8–P11 old mice. Suppression of EPSCs was measured at three concentrations of EGTA-AM (50, 100 and 200 μM), and the data was fitted with a Hill equation, indicating a half maximal effective concentration of 87 μM. (**D1–D2**) Same as (**A1-A2**), but now for MNTB neurons recorded in adult mice (P60–P100). Note that similar to the measurements at P8–P11, EPSCs were strongly (~80%) blocked by acute application of 200 μM EGTA-AM. (**E, F**) An example of EPSC suppression by 100 μM EGTA-AM in an adult synapse (**E**), and the dose-response relation measured at 50, 100, and 200 μM EGTA-AM, which indicated a half maximal effective concentration of 92 μM EGTA-AM. Note the similar concentration-dependent effects of EGTA-AM in immature (**A-C**), and adult synapses (**D-F**).

Finally, we wished to investigate the EGTA sensitivity of transmitter release, and its dependence on the developmental state of the calyx of Held synapse. We used the membrane-permeable EGTA analog, EGTA-AM, which allowed us to investigate synapses from adult mice (P60–P100) not amenable to paired recordings. We first investigated immature synapses (P8–P11), and found a gradual suppression of EPSC amplitudes upon acute application of 200 μM EGTA-AM (Fig 8A1 and 8A2). After applications times of 15–20 minutes, EPSC amplitudes were suppressed to 16 ± 7% of their control value (n = 2). Using lower concentrations of EGTA-AM (100, and 50 μM), the suppression of EPSCs was smaller, which demonstrates a concentration-dependent effect of EGTA-AM with an apparent half-maximal effective concentration of 87 μM (Fig 8B and 8C). These results agree with earlier findings showing that low millimolar concentrations of EGTA, or 200 μM EGTA-AM suppress transmitter release at the immature rat [18,52] and mouse [12] calyx of Held.

We then investigated adult synapses from P60–P100 mice using the same experimental approach. Acute application of 200 μM EGTA-AM again strongly suppressed EPSC amplitudes, with 20.1 ± 5% of the control EPSC amplitude remaining (Fig 8B1 and 8B2). This value was slightly larger than the remaining fraction of the EPSC at the young calyx synapse, but the difference did not reach statistical significance (p = 0.6). The dose-dependency of the steady-state block of EPSCs by EGTA-AM indicated a half-maximal effective concentration of 92 μM (Fig 8F), similar to the value found at young calyx synapses. These results show that overall, ultrafast transmitter release at the calyx synapses remains sensitive to EGTA-AM, although its efficiency might be slightly smaller in mature mice, in agreement with recent findings [53]. Therefore, the measurements of Ca^2+^ current release cooperativity (Fig 7) and of the EGTA-AM sensitivity (Fig 8) suggest that despite a developmental tightening of the Ca^2+^ channel—release coupling (Fig 7; [12,20–22], domain overlap remains relevant for the mature calyx of Held synapse. This also suggests that while some fast-releasing synapses employ single channel release control [54–56], other fast-releasing synapses like the calyx of Held can rely on release control by several Ca^2+^ channels.

## Discussion

We developed an active zone model with several docked vesicles and multiple Ca^2+^ channels at realistic densities, to explore Ca^2+^ channel localizations compatible with domain overlap control of release. Although high-resolution information on the localization of vesicles, and in some cases Ca^2+^ channels has become available in recent years [25,26,28,53,57], there is still little information on the spatial co-localization of vesicles and Ca^2+^ channels on the nanometer scale, especially for CNS synapses. We started by analyzing the x-y distribution of docked vesicles in a sample of serially reconstructed active zones from calyx of Held synapses, with the aim to derive constraints on where Ca^2+^ channels might be positioned. We found that the position of docked vesicles was not significantly different from random spatial distributions, and the average density of docked vesicles at the active zone was high. Nevertheless, void spaces were apparent, which might be occupied by Ca^2+^ channels.

We then used stochastic MCell modelling at example active zones to explore where multiple Ca^2+^ channels might be positioned to reproduce salient features of domain overlap control of release. A first insight was that randomly distributed Ca^2+^ channels at a realistic density were unable to predict a high Ca^2+^ current—release cooperativity, and produced exceedingly high release probability. This indicates that for release control by multiple channels, the channels must be localized at some distance from docked vesicles. To implement a model of release control of multiple docked vesicles by multiple Ca^2+^ channels with the smallest possible number of free parameters, we then explored two simple rules for the distribution of Ca^2+^ channels, the exclusion zone model and the supercluster model; the latter was inspired by a previous theoretical work [11]. There might be other possible arrangements of Ca^2+^ channels at the active zone; for example, Ca^2+^ channels might be localized in several small clusters. It is likely, however, that also in this case Ca^2+^ channels would need to obey a certain distance from docked vesicles, and that sub-clusters of Ca^2+^ channels would likely be positioned in the void spaces at some distance from docked vesicles. Therefore, an arrangement with several Ca^2+^ channel sub-clusters is overall similar to the exclusion zone arrangement.

### Heterogeneity of release probability at single active zones

The supercluster model and the exclusion zone model predicted values of Ca^2+^ current—release cooperativity in the range of 2.8–3.3 (Figs 3–5). The maximal value of this parameter predicted by Ca^2+^ sensor models with 5 Ca^2+^ ion binding sites [7,8] is ~ 3.5, as shown by previous simulations with AP-like stimuli [11,42]. Therefore, both models preserve the high intrinsic Ca^2+^ cooperativity nearly completely when the number of open Ca^2+^ channels is varied, as expected for domain overlap control of release [9–13]. Since the Ca^2+^ current—release cooperativity was therefore not a distinguishing feature between the two Ca^2+^ channel arrangements, our further analysis concentrated on the heterogeneity of p_ves_ between vesicles.

Our model allowed us to track the individual release probabilities of multiple docked vesicles, an approach which received little attention in previous modeling studies. We assumed that all docked vesicles belonged to the readily-releasable pool consistent with experimental considerations (see discussion in [29]). Furthermore, we assumed that no mechanism exists which would limit release to single fusion events at a given active zone (thus, no "lateral inhibition" mechanism as proposed in refs. [58,59]). For simplicity, we also assumed that all vesicles have the same Ca^2+^ sensitivity; thus, our model would be unable to describe the biphasic release response upon Ca^2+^ uncaging [47]. In the exclusion zone model with a distance of 30 nm which yielded a realistic average p_ves_ of ~ 0.1, we observed mostly single or no release events at the small active zone, whereas multiple fusion events ("multivesicular release") were rare (Fig 3C, *bottom*). Therefore, we think that no special depression mechanism needs to be postulated to explain that under normal conditions, there is a low degree of multivesicular release [60].

We found that the two Ca^2+^ channel arrangements could mainly be distinguished by the degree of heterogeneity of release probability. The large heterogeneity of p_ves_ produced by the supercluster model is expected, because placing all Ca^2+^ channels in a small spot inevitably produces strong gradients of local [Ca^2+^]_i_ across an active zone, since active zones can extend over a few hundred nanometers. Furthermore, docked vesicles likely create a hindrance for Ca^2+^ diffusion towards the more distant vesicles (Figs 4A and 5D). Our modelling approach included such a "shading" effect of Ca^2+^ diffusion by docked vesicles (see Materials and Methods).

To further distinguish between the two Ca^2+^ channel arrangements, more insights into the heterogeneity of p_ves_ amongst vesicles within individual active zones must be obtained. Earlier work using optical methods has shown a substantial variability of release probabilities [61], but this heterogeneity likely represents differences across active zones. At the calyx synapse, prolonged presynaptic depolarizations have been used to estimate release probability heterogeneity [45–47]. Some studies concluded that SRP release with time constants of ~ 20 ms is caused by longer distances of SRP vesicles to Ca^2+^ channels (the "positional" model; ref. [45,46]). We modelled the response of the supercluster- and exclusion zone models to prolonged voltage-clamp depolarizations (Fig 6). As expected, the supercluster arrangement produced a larger degree of heterogeneity in the release times than the exclusion zone model. However, even in the supercluster arrangement at the large active zone, the spread in release delays was in the range of only 2–3 ms, too small to explain SRP release with time constants of ~ 20 ms [45–47]. We conclude that the separation of FRP and SRP release cannot be used to inform about the validity of the supercluster versus the exclusion zone arrangement. The inability of realistic active zone models to predict slow SRP release with time constants ~ 20 ms under positional assumptions is consistent with the earlier view that the slow release phase is caused by an intrinsic mechanism [47].

### Constraints from ultrastructural data on the localization of Ca^2+^ channels

To distinguish between the supercluster and exclusion zone arrangements, it will also be necessary to obtain more ultrastructural data on the exact localization of Ca^2+^ channels at active zones of CNS synapses. At hippocampal synapses, freeze-fracture replica labeling has shown several loosely organized, stripe-like clusters of particles detected by an antibody against Ca_V_2.1 (P/Q-type Ca^2+^ channel) (see Figs 6–8 in ref. [28]). The hypothetical distributions of Ca^2+^ channels produced by the exclusion zone (Fig 3A and 5A) could correspond to such stripe-like clusters of Ca_V_2.1 particles observed in the previous study [28].

A recent study obtained evidence for the localization of P/Q-type Ca^2+^ channels from freeze-fracture labeling at the calyx of Held synapse. Combined with functional findings on the inhibition of release by EGTA, a release model for the calyx synapse was derived [53]. The study found clusters of Ca^2+^ channels; a cluster was defined if particles were located within 100 nm distance. Example images show that Ca_V_2.1 particles can be located over quite long distances (~ 100–300nm; see e.g. Fig 1C and 6H of ref. [53]). Modelling then suggested that docked vesicles must be localized at a distance of 20–30 nm from a somewhat arbitrary Ca^2+^ channel cluster of ~ 100 nm diameter, to explain the EGTA-sensitivity of release [53]. Our finding of an exclusion zone distance of 30 nm is consistent with this previous distance estimate. Nevertheless, such distance estimates from modelling are subject to parameters which are only incompletely known, including the Ca^2+^ channel density [41] and the kinetics, concentration and mobility of endogenous Ca^2+^ buffers (see S2 Text), and should therefore be taken with care.

Based on our computational model, and on the placement of Ca^2+^ channels at active zones shown so far in EM studies [28,53], we find it unlikely that all Ca^2+^ channels should be localized in a single cluster ("supercluster") within an active zone, as predicted by a previous modelling study [11]. Rather, we regard it as more likely that Ca^2+^ channels are placed either randomly, or else in several sub-clusters in the void spaces in-between docked vesicles, but with a certain minimal distance to docked vesicles.

### Possible mechanism enabling an exclusion zone

Ion channels are mobile in membranes as has been found for postsynaptic glutamate receptors [62]; however, the mobile properties of presynaptic Ca^2+^ channels are less well known [63]. In addition, docked vesicles might also undergo lateral movement at synapses. An exclusion zone of several tens of nanometer around docked vesicles might be caused by a hindered mobility of Ca^2+^ channels close to the docked vesicle, maybe caused by the multitude of vesicle-near proteins which make up the docking complex and the vesicle fusion machinery [64,65]. In addition, specific proteins likely regulate the minimal distance between vesicles and Ca^2+^ channels. At the neuromuscular synapse, rib-like proteins have been found which likely hold Ca^2+^ channels at a certain distance from vesicles [66], and filamentous structures playing similar roles might also be present at CNS synapses [25]. One candidate for such a structure at CNS active zones is the filamentous protein Septin-5. When this protein was genetically inactivated, an *increased* release probability and a *tighter* spatial coupling resulted [67], which suggested that Septin-5 normally keeps Ca^2+^ channels at a certain distance from docked vesicles. During developmental maturation, the Ca^2+^ channel—vesicle coupling distance becomes tighter (Fig 7) [12,20–22], which probably also requires protein re-arrangements to specifically regulate the minimal Ca^2+^ channel—vesicle distance with a precision of ~ 10 nm or less. Scaffold proteins at the active zone which regulate Ca^2+^ channel vesicle coupling distance should be investigated in future studies.

## Materials and Methods

### Ethics statement

Procedures of mouse breeding, handling, and the sacrification of mice before slice preparation were approved by the Veterinary Office of the Canton of Vaud, Switzerland (authorization no. 2063.3).

### Analysis of docked vesicle distribution

The sample of the location of docked vesicles at n = 15 active zones is the same as that previously published by [29] (n = 15 active zones from a P11 control mouse). In brief, serial transmission EM images were taken from 10–20 subsequent thin sections (50 nm), which were made following standard fixation and resin embedding procedures. The images were aligned, and pre- and postsynaptic membranes and vesicles were drawn in each section using the TrackEM2 program [68] of the FIJI software [69], and the extent of the PSD was annotated. Only active zones that were completely contained in the series were maintained in the final data set. Each active zone was 3D- reconstructed by the FIJI software, and the nearest distance between vesicle membrane and active zone membrane was measured in 3D, and plotted. Vesicles at 10 nm or less showed a distinct peak in these distance distributions [29], and were regarded as the pool of "docked" vesicles.

In order to map the x-y location of docked vesicles, we unfurled the curved surface of the active zone with its attached docked vesicles, using a Matlab program. The shortest distance from the vesicle membrane to the active zone membrane was calculated in the 3D model, and the corresponding point in the flat surface was taken as the remapped vesicle position.

### Model implementation, analysis and statistics

We used the Monte Carlo simulator MCell [70] to track the fate of individual Ca^2+^ ions that entered through various individual Ca^2+^ channels. The simulator was run on a Blue Gene/Q system, and the MCell based model is available on ModelDB (http://senselab.med.yale.edu/modeldb/). In general, 2000–4000 repetitions were run to determine an average "release response" for a given simulated stimulus, which is the release rate for all individual vesicles in response to one stimulus; see e.g. Fig 3B, *bottom*). This simulation was then repeated for each AP width (usually, n = 9 different AP widths), to construct a given dose-response curve of release rates versus Ca^2+^ influx (e.g. Fig 2E, *bottom*). The dose-response curve was fitted with a line in double-logarithmic coordinates to obtain the "Ca^2+^ current—release cooperativity" (see main text for definition). The highest datapoints were usually excluded from the fit range, since they deviated from the steepest slopes, as expected from the beginning saturation of the Ca^2+^ sensor for vesicle fusion > 10 μM [Ca^2+^]_i_. In addition, the lowermost data points at very low release probabilities were often inaccurate despite the number of individual repetitions (2000–4000, see above); in this case, the lowermost data points were not included in the fit range. In order to evaluate the statistical robustness of the slope estimates, the entire simulation was repeated 3–5 times, using different random seeds for the spatial arrangements of Ca^2+^ channels; these repetitions are referred to as "independent spatial seeds" in the main text.

### Model parameters

The simulated compartment measured 600 nm by 600 nm and had a height of 1200 nm. Ca^2+^ was reflected off the walls; the size of the compartment was chosen based on published nearest-neighbor distances of active zones at the immature rat calyx of Held [71]. Models contained Ca^2+^ channels, endogenous Ca^2+^ buffers and Ca^2+^ sensors for release. Docked vesicles were present as spheres with 45 nm diameter inaccessible to Ca^2+^ ions. Ca^2+^ channels opened and closed stochastically during the AP according to a two-state model (Fig 2A). Ca^2+^ channels had a realistic single channel current of 0.12 pA at 0 mV [31,41]. The standard AP had a half-width of 0.49 ms; the peak open probability of Ca^2+^ channels was ~ 0.6 (Fig 2D). Ca^2+^ diffusion away from individual channels was modelled stochastically with MCell. Two mobile Ca^2+^ buffers, ATP and Parvalbumin, and an immobile (fixed) buffer were present in the simulations, with parameters as given in S1 Table. The Ca^2+^ sensor was a kinetic model of Ca^2+^ binding and vesicle fusion, with parameters which reflect extensive previous Ca^2+^ uncaging studies at the calyx of Held synapse. The specific kinetic model was a 5-site model (Fig 2A), with parameters as reported in ref. [37]. See S2 Text for further justification of the model parameters.

### Electrophysiology

Transverse brain slices on the level of the medial nucleus of the trapezoid body were prepared from C57Bl6 mice of three different age groups: P8–P11; P15–P16, and P60–P100 ("adult"). Mice were killed by decapitation, sometimes following a brief isoflurane anesthesia in the oldest age group, in a protocol approved by the Veterinary office of the Canton of Vaud, Switzerland. Transverse brain slices of 200 μm thickness were made with a vibratome (VT1000 or VT1200; Leica Microsystems, Wetzlar, Germany), and stored in a submerged keeping chamber containing a standard bicarbonate buffered solution (for composition, see below), bubbled with 95% O_2_ and 5% CO_2_. For paired pre- and postsynaptic recordings (Fig 6), the extracellular solution contained 50 μM D-APV, 100 μM cyclothiazide (CTZ), 1 μM tetrodotoxin (TTX; all from BIOTREND, Wangen, Switzerland) and 10 mM TEA-Cl (Sigma Aldrich/Fluka, Buchs, Switzerland), to suppress unwanted current components and AMPA-receptor desensitization (CTZ). Series resistances (R_s_) were in the range of 3–10 MΩ and 5–25 MΩ for post- and presynaptic recordings, and were compensated by up to 80 and 50% for post- and presynaptic recordings respectively, using the patch-clamp amplifier (EPC10/2; HEKA Electronik, Lambrecht/Pfalz, Germany). For postsynaptic recordings, the remaining R_s_ error was compensated offline. Brief steps to +60 mV of various lengths were applied from a holding potential of -70 mV, resulting in the recruitment of an increasing number of Ca^2+^ channels giving rise to Ca^2+^ "tail" currents during the repolarization phase (Fig 6A). In fiber stimulation experiments (Fig 8), afferent fibers were stimulated by a custom-made bipolar stimulation electrode placed close to the midline of the slice, using 0.2 ms long pulses of 1–10 V amplitude from an isolated stimulation unit (ISO-STIM01D-100, NPI electronic, Tamm, Germany). EGTA-AM (Life Technologies, Zug, Switzerland) was applied by bath perfusion, using stock solutions of 200 mM (in DMSO, Sigma), at the indicated final concentrations (50, 100 or 200 μM). Stocks of EGTA-AM solutions in DMSO were kept desiccated at–20°C for a maximum of 3 months.

The extracellular solution contained (in mM): 125 NaCl, 2.5 KCl, 25 NaHCO_3_, 1.25 NaH_2_PO_4_, 25 glucose, 1 MgCl_2_, 2 CaCl_2_, 0.4 ascorbic acid, 3 myo-inositol and 2 Na-pyruvate, continuously bubbled with 95% O_2_/5% CO_2_ (pH 7.4). The intracellular (pipette) solution for pre- and postsynaptic recordings contained (in mM): 130 Cs-gluconate, 20 TEA-Cl, 10 HEPES, 5 Na_2_-phosphocreatine, 4 MgATP, 0.3 Na_2_GTP (pH 7.2 adjusted with CsOH). For pre- or postsynaptic recordings, this solution was supplemented with 0.1 or 5 mM EGTA, respectively. All chemicals were from Sigma Aldrich/Fluka (Buchs, Switzerland) unless indicated.

## Supporting Information

S1 Text Ripley’s K-Function. (DOCX)

S2 Text Detailed justification of model parameters. (DOCX)

S1 Table The most important parameters of the active zone model. (DOCX)
