# Supplementary material for: An Exclusion Zone for Ca2+ Channels around Docked Vesicles Explains Release Control by Multiple Channels at a CNS Synapse
Source: PLoS Comput Biol. 2015 May 7;11(5):e1004253. doi: 10.1371/journal.pcbi.1004253 (PMC4423980; doi:10.1371/journal.pcbi.1004253)
Supplement: S1 Text — (DOCX) [file pcbi.1004253.s001.docx]

Supporting Information for:

**An exclusion zone for Ca^2+^ channels around docked vesicles explains release control by multiple channels at a CNS synapse**

Daniel Keller, Norbert Babai, Olexiy Kochubey, Yunyun Han, Henry Markram, Felix Schürmann, Ralf Schneggenburger

**S1 Text. Ripley’s K Function**

We performed an K- function analysis of vesicle distribution within the active zone. For a given test radius r and test vesicles i and j, we use an indicator function k(i,j) = 1/(fraction of the circumference of a circle centered on i and passing through j that lies within the study area). This is necessary because not all circles of radius r will fall within the allowed active zone boundaries. The K function was calculated according to:

$K\left( r \right)=\lambda^{-1}\sum_{i\neq j} k(i,j)/N$,

where the average density of points λ=N/A_AZ_. L(r) could then be calculated according to the equation:

$$L\left( r \right)=\sqrt{\frac{K(r)}{\pi}}$$

Significance tests of r-L(r) were done using Monte Carlo methods. We calculated this value from 500 spatial seeds in which vesicles were randomly placed in each the active zone, and estimated the 99% confidence interval (99% CI) as being the 99th percentile highest value obtained (Prior et al., 2003).

**References**

Prior IA, Muncke C, Parton RG, Hancock JF (2003) Direct visualization of Ras proteins in spatially distinct cell surface microdomains. J Cell Biol 160: 165-170.
