# Supplementary material for: An Exclusion Zone for Ca2+ Channels around Docked Vesicles Explains Release Control by Multiple Channels at a CNS Synapse
Source: PLoS Comput Biol. 2015 May 7;11(5):e1004253. doi: 10.1371/journal.pcbi.1004253 (PMC4423980; doi:10.1371/journal.pcbi.1004253)
Supplement: S2 Text — (DOCX) [file pcbi.1004253.s002.docx]

Supporting Information for:

**An exclusion zone for Ca^2+^ channels around docked vesicles explains release control by multiple channels at a CNS synapse**

Daniel Keller, Norbert Babai, Olexiy Kochubey, Yunyun Han, Henry Markram, Felix Schürmann, Ralf Schneggenburger

**S2 Text. Detailed Justification of model parameters**

*Single Ca^2+^ channel current*. Since the single channel Ca^2+^ current (i_Ca_) is an important variable in setting the microdomain [Ca^2+^]_i_ close to an open channel [16], we carefully screened the literature results regarding i_Ca_. Weber et al. 2010 (ref. [31]) showed that L-type currents had i_Ca_ of ~ 0.13 at 0 mV and 2 mM [Ca^2+^]. Variance-mean analysis of presynaptic Ca^2+^ currents at mossy fiber boutons estimated i_Ca_ with 0.13 pA at 0 mV and physiological [Ca^2+^] (ref. [30]), whereas the corresponding value at the calyx of Held was slightly lower (0.08 pA; ref. [32]). Finally, a recent paper using cell-attached presynaptic recordings estimated the single channel current at 0 mV and 10 mM [Ca^2+^] with 0.27 pA [41]. Considering a two-fold increase in i_Ca_ between 2 and 10 mM [Ca^2+^] ([Church and Stanley, 1996](#_ENREF_2); [Schneggenburger et al., 1999](#_ENREF_7)), the latter value corresponds to 0.13 pA. Thus, the value of 0.12 pA used here is justified by several recent direct experimental estimates [30-32, 41]. We assumed a linear voltage dependence of the single channel current, with a slope of 2.2 pS.

*Ca^2+^ channel gating and AP waveform.* We adapted a Hodgkin-Huxley Ca^2+^ channel model [19] to its direct stochastic counterpart (Fig. 2A). The channel possesses open and closed states, with transitions between them governed by voltage dependent rates. To drive the channels, we fitted the AP recorded at the calyx of Held nerve terminal [19] to a Gaussian function with skew. This "standard AP" departed from -80 mV, had a peak amplitude of +28 mV and a half-width of 0.49 ms (Fig. 2D, *top*; black trace). To model the effect of recruiting more, or fewer Ca^2+^ channels, we either prolonged, or shortened the APs (Fig. 2D).

*Ca^2+^ sensor*. We used a "five site" model of Ca^2+^ binding and vesicle fusion, as determined experimentally in direct presynaptic Ca^2+^ uncaging studies at the calyx of Held synapse [8, 22, 36, 37], using the parameters reported in [37]. At the calyx of Held, many published Ca^2+^ uncaging studies are available [7, 8, 21, 22, 36 - 39]. The studies agree that vesicle fusion is triggered with a high intrinsic Ca^2+^ cooperativity of release (slope of ~ 4) in a range of ~ 2 - 10 µM [Ca^2+^]_i_, requiring the use of kinetic models with at least 5 Ca^2+^ ions binding to a release sensor. Some studies additionally investigated Ca^2+^ control of release at [Ca^2+^]_i_ < 2 µM, and showed that at low [Ca^2+^]_i_, the dose-response curve was less steep, with a slope as low as 0.5 close to resting [Ca^2+^]_i_ [38, 39] ([Kochubey and Schneggenburger, 2011](#_ENREF_4)). Nevertheless, the "5-site" model of Ca^2+^ binding and vesicle fusion [8] should be a valid approximation of the Ca^2+^ regulation of transmitter release during AP-like Ca^2+^ influx, which causes microdomain [Ca^2+^]_i_ signals clearly above 3 µM (Fig. 3B). Importantly, two Ca^2+^ uncaging studies at the calyx synapse found that the intrinsic Ca^2+^ sensitivity of vesicle fusion was not changed significantly with development in rats [22], or was only slightly rightward shifted with development in mice [21]. Therefore, the Ca^2+^ sensitivity of vesicle fusion has been measured and validated by a series of Ca^2+^ uncaging studies at the calyx of Held synapse.

*Intracellular Ca^2+^ buffering*. Cells contain endogenous fixed Ca^2+^ buffer substances with a buffer capacity κ_S_ of at least ~ 30 [16]; κ_S_ was found to be ~ 40 at the calyx of Held [33]. Spike-like manipulations of [Ca^2+^]_i_ with pulsed laser uncaging combined with model calculations have suggested a K_d_ ≥ 10 µM, and an on-rate k_on_ of ≥ 1.0×10^8^ M^-1^s^-1^ for the endogenous buffer [35]; these values were used here (see S1 Table). Given that κ_S_ = 40 at the calyx of Held [33], a concentration [S] = 400 µM of endogenous buffer was used, since κ_S_ ≈ [S]/K_d_ (ref. [16]). We found that a limited diffusional mobility of the endogenous Ca^2+^ buffer (diffusion coefficient D = 1·10^-7^ cm^2^ s^-1^) was beneficial for speeding the decay of the [Ca^2+^]_i_ transients (not shown). However, for all simulations shown here (Figs 2 - 6), we assumed that the endogenous buffer was immobile. ATP, a mobile Ca^2+^ buffer [5], was present at a total concentration of 2 mM in all simulations. Initial simulations showed that without adding ATP, the estimated exclusion zone distance for obtaining p_ves_ of 0.1 was larger (40 nm; results not shown). Finally, we assumed the presence of a Parvalbumin (PV)-like Ca^2+^ buffer with concentration of 50 µM as found experimentally at the calyx synapse [34]; we assumed a diffusional mobility D = 1·10^-6^ cm^2^ s^-1^ for PV (S1 Table). Nevertheless, due to its slow Ca^2+^ binding kinetics and low concentration, PV did not notably influence the simulated [Ca^2+^]_i_ transients relevant for fast release.

*Number and distribution of Ca^2+^ channels in the "realistic" active zone model*: We modelled release at two selected active zones (#5, and #11 in Fig. 1A); the observed density of docked vesicles at these active zones was 100 and 125 ves / µm^2^, close to the average value (see Fig. 1C; arrows). For both active zones, we assumed a Ca^2+^ channel density of ~ 280 / µm^2^. This value is consistent with the reported average value of ~ 240 gold particles / µm^2^, when Ca^2+^ channels were detected in freeze fracture replica labelling EM of hippocampal active zones [28]. This density value yielded n = 14 and n = 25 Ca^2+^ channels for the two example active zones. A recent study using cell-attached recordings at presumed single active zones from calyx of Held nerve terminals, has reported a somewhat larger average number (42 ± 2 channels; ref. [41]. Nevertheless, this value might be an upper limit, since it remains possible that there was a sampling bias towards larger active zones, and that in some cases several active zones were present in the cell-attached recordings.

After we fixed the Ca^2+^ channel density to 280 / µm^2^, we only modified a single distance parameter, the exclusion zone distance (Fig. 3A, D), or the distance between the supercluster and the nearest vesicle (Figs 3F, 4D).

The most important model parameters are listed in S1 Table.

**References**

**(those not cited in the main text)**

Church, P.J., and Stanley, E.F. (1996). Single L-type calcium channel conductance with physiological levels of calcium in chick ciliary ganglion neurons. J Physiol 496, 59-68.

Kochubey, O., and Schneggenburger, R. (2011). Synaptotagmin increases the dynamic range of synapses by driving Ca^2+^ - evoked release and by clamping a near-linear remaining Ca^2+^ sensor. Neuron 69, 736-748.

Schneggenburger, R., Meyer, A.C., and Neher, E. (1999). Released fraction and total size of a pool of immediately available transmitter quanta at a calyx synapse. Neuron 23, 399-409.
