# Supplementary material for: An Exclusion Zone for Ca2+ Channels around Docked Vesicles Explains Release Control by Multiple Channels at a CNS Synapse
Source: PLoS Comput Biol. 2015 May 7;11(5):e1004253. doi: 10.1371/journal.pcbi.1004253 (PMC4423980; doi:10.1371/journal.pcbi.1004253)
Supplement: S1 Table — (DOCX) [file pcbi.1004253.s003.docx]

Supporting Information for:

**An exclusion zone for Ca^2+^ channels around docked vesicles explains release control by multiple channels at a CNS synapse**

Daniel Keller, Norbert Babai, Olexiy Kochubey, Yunyun Han, Henry Markram, Felix Schürmann, Ralf Schneggenburger

**S1 Table. The most important parameters of the active zone model.**

| ***Geometrical parameters*** |  |
| --- | --- |
| width of simulated space between AZs | 0.6 μm |
| height of simulated volume | 1.2 μm |
| ***Parameters of standard AP waveform*** |  |
| resting membrane potential, V_m_ | -80 mV |
| peak V_m_ | +28 mV |
| AP half-width | 0.49 ms |
| ***Release Model*** |  |
| binding sites | 5 |
| cooperativity factor | 0.25 |
| k_on_ | 1.19·10^8^ M^-1^·s^-1^ |
| k_off_ | 8745 s^-1^ |
| final vesicle fusion rate, γ | 6995 s^-1^ |
| ***Calcium Channel Model*** |  |
| single channel conductance | 2.2 pS, linear |
| single channel current at 0mV | 0.12 pA |
| α_m_ | α_0_·exp(V/V_α_), α_0_ =1.78 ms^-1^, V_α_ =23.3 mV |
| β_m_ | β_0_·exp(-V/V_β_), β_0_=0.14 ms^-1^, V_β_=15.0 mV |
| ***Ca^2+^ diffusion and - buffering*** |  |
| resting [Ca^2+^]_i_ | 70 nM |
| D_Ca_^2+^ | 2.2·10^-6^ cm^2^·s^-1^ |
| *Endogenous Ca^2+^ Buffer 1 (immobile)* |  |
| buffer capacity κ_s_ | 40 |
| concentration | 400 μM |
| K_d_ | 10 μM |
| k_on_ | 10^8^ M^-1^s^-1^ |
| *Endogenous Ca^2+^ Buffer 2 (Parvalbumin)* |  |
| concentration | 50 μM (100µM of single Ca^2+^-binding site) |
| k_on_, _Ca_^2+^ | 4·10^8^ M^-1^·s^-1^ |
| k_off_, _Ca_^2++^ | 4 s^-1^ |
| k_on_, _Mg_^2+^ | 10^6^ M^-1^·s^-1^ |
| k_off_, _Mg_^2+^ | 30 s^-1^ |
| Mg^2+^ basal concentration | 0.3 mM |
| D_PV_ | 10^-6^ cm^2^·s^-1^ |
| *Endogenous Ca^2+^ Buffer 3 (ATP)* |  |
| K_d, Ca_^2+^ | 90 μM |
| K_d, Mg_^2+^ | 45 μM |
| k_on_, _Ca_^2+^_, Mg_^2+^ | 5·10^8^ M^-1^s^-1^ |
| concentration | 2 mM |
| D_ATP_ | 2.2·10^-6^ cm^2^·s^-1^ |
| *Exogenous Ca^2+^ Buffer 4 (EGTA)* |  |
| K_d, Ca_^2+^ | 70 nM |
| k_on_, _Ca_^2+^ | 10^7^ M^-1^s^-1^ |
| D_EGTA_ | 2.2·10^-6^ cm^2^·s^-1^ |
